# Conservative therapy of epidural hematoma with atorvastatin combined with glucocorticoids: cases report and literature review

**DOI:** 10.3389/fsurg.2025.1587988

**Published:** 2025-04-30

**Authors:** Chenrui Wu, Yu Tian, Ruichen Zhao, Runfang Chen, Chuanlin Xu, Jinsheng Huang, Rongcai Jiang

**Affiliations:** ^1^ Department of Neurosurgery, Xuanwu Hospital, Capital Medical University, Beijing, China; ^2^ Department of Neurosurgery, Sichuan Provincial People's Hospital, University of Electronic Science and Technology of China, Chengdu, China; ^3^ Department of Neurosurgery, Tianjin Neurological Institute, State Key Laboratory of Experimental Hematology, Laboratory of Post-Neuroinjury Neurorepair and Regeneration in Central Nervous System Tianjin & Ministry of Education, Tianjin Medical University General Hospital, Tianjin, China; ^4^ Department of Neurosurgery, Nanping First Hospital Affiliated to Fujian Medical University, Fujian, China

**Keywords:** epidural hematoma, conservative treatment, atorvastatin, dexamethasone, meningeal lymphatic system

## Abstract

Epidural hematomas (EDH), typically requiring surgery, may be managed conservatively in select patients. We investigated atorvastatin (10 mg/day) combined with dexamethasone (2.25 mg/day) as conservative therapy in six EDH patients (GCS ≥ 13, volume < 30 ml) post-trauma. All patients recovered fully without surgery, and literatures support conservative care for stable EDH. Our findings suggest this combination therapy may promote hematoma absorption. In conclusion, atorvastatin/dexamethasone shows promise as a non-surgical EDH option, warranting further investigation.

## Introduction

Epidural hematoma (EDH) is a critical neurosurgical condition characterized by the accumulation of blood between the dura mater and the skull's inner surface. It typically results from traumatic injury leading to skull fractures, the laceration of the middle meningeal artery, or dural venous sinuses. EDH accounts for approximately 1%–3% of all head injuries and is most prevalent in young adults due to high-impact trauma such as traffic accidents or falls (1, 2). The classical clinical presentation includes a brief loss of consciousness followed by a lucid interval and subsequent rapid neurological deterioration if left untreated.

The standard management of EDH has traditionally been a prompt surgical evacuation, especially in patients presenting with hematomas greater than 30 cm³ in volume, thickness exceeding 15 mm, or midline shift greater than 5 mm, as these factors are associated with increased morbidity and mortality (1). However, emerging evidence challenges this paradigm, demonstrating that select patients—particularly those with preserved neurologic function (GCS ≥ 14) and stable radiographic parameters—may achieve favorable outcomes through conservative strategies (3, 4). Studies have shown that small to moderate-sized hematomas without significant mass effects or neurological deficits may resolve spontaneously under careful observation (5, 6).

Conservative treatment strategies aim to minimize the risks associated with surgery, such as infection, hemorrhage, and anesthesia-related complications, while relying on the body's physiological mechanisms to reabsorb the hematoma. Recent literature has provided evidence supporting conservative management in carefully selected cases of EDH, emphasizing the importance of patient selection criteria, including hematoma size, location, and the patient's neurological status (4, 7).

Parallel to these developments, pharmacological interventions have emerged as potential adjuncts in managing cranial hematomas. Notably, atorvastatin, a lipid-lowering agent in the statin class, has demonstrated therapeutic benefits beyond its cholesterol-lowering effects. Atorvastatin possesses anti-inflammatory, anti-apoptotic, and anti-angiogenic properties, which have been investigated in chronic subdural hematoma (CSDH) (8). In addition, dexamethasone has been used in the conservative management of CSDH since the 1970s (9), and we found an enhanced anti-inflammatory effect of the combination of atorvastatin and dexamethasone in preclinical trials (10). Therefore, we tried the combination regimen of atorvastatin and dexamethasone in the clinical patients.

In patients with CSDH, atorvastatin has been shown to promote hematoma resolution and improve neurological outcomes. The underlying mechanisms are thought to involve the upregulation of angiogenic factors such as vascular endothelial growth factor (VEGF), enhancement of endothelial progenitor cell (EPC) mobilization, and suppression of inflammatory cytokines, all contributing to improved vascular stability and hematoma absorption (10). Our recent research has demonstrated that atorvastatin can effectively treat CSDH by enhancing lymphatic drainage (11).

Given the pathophysiological similarities between CSDH and EDH—particularly the role of inflammation and lymphatic drainage in hematoma persistence—it is plausible that atorvastatin could benefit patients with EDH. These findings have opened new avenues for non-surgical treatment approaches in select patients with EDH.

In this report, we have explored the role of atorvastatin in the conservative management of EDH. We report a series of six patients who presented with epidural hematomas and were treated conservatively with oral atorvastatin. All patients achieved full recovery without the need for surgical intervention. To our knowledge, this is the first study to evaluate the efficacy of atorvastatin in managing EDH.

## Case series

### Case presentation

**Figure 1:**
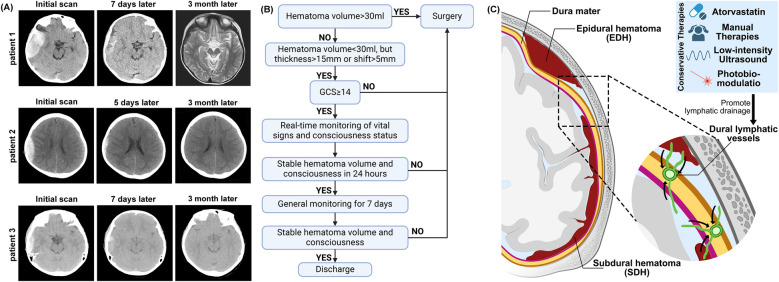
**(A)** Representative patients head CT shows changes in hematoma at admission and after the treatment. **(B)** Clinical decision diagram for the treatment of EDH patients. **(C)** The diagram illustrates the pathological anatomy of EDH and SDH. Targeting the dura mater lymphatic vessels may be a potential approach for treating EDH and SDH.

From January 2023 to January 2025, we collected data on six patients with epidural hematomas caused by high falls or motor vehicle accidents (MVA) at Tianjin Medical University General Hospital, Henan Province Kailuan General Hospital, Fujian Province Nanping First Hospital, and Beijing Xuanwu Hospital. The patients ranged from 8-year-old children to 62-year-old adults with a history of acute trauma within 3 days. The average hematoma thickness was 13.5 ± 6.6 mm, the average hematoma volume was 15.4 ± 9.3 ml, and the average midline shift was 3.0 ± 3.2 mm. All patients had a good or slightly impaired consciousness state (GCS ≥ 13) upon admission and immediately underwent CT scans to confirm the location and size of the hematomas. The hematoma volume for all patients was less than 30 ml, but one had a midline shift of more than 5 mm, and three patients had hematoma thickness greater than 15 mm (Figure 1A and Table 1). Due to the patients' stable consciousness, they received conservative treatment with atorvastatin and dexamethasone, along with monitoring of vital signs within 3 days after the injury.

**Table 1:** Clinical patient information of the present report.

Age/Sex	Mechanism of injury	Hematoma thickness (mm)	Hematoma volume (ml)	Midline shift (mm)	Initial neurological state (GCS)	Prognosis (GOS)
62/Female	Fall	16	17.9	4	14	Good recovery
9/Male	MVA	23	28.6	8	13	Good recovery
10/Female	MVA	6	4.3	0	15	Good recovery
21/Male	MVA	8	6.8	0	15	Good recovery
8/Male	MVA	18	22.1	5	14	Good recovery
37/Male	MVA	10	12.5	1	15	Good recovery

MVA, Motor Vehicle Accident; GCS, Glasgow Coma Scale; GOS, Glasgow Outcome Scale.

### Treatment protocol

All patients were treated with a combination of atorvastatin and dexamethasone, aiming to promote the resolution of the hematoma and reduce inflammation. Atorvastatin, known for its anti-inflammatory and angiogenic properties, was administered to enhance lymphatic drainage and facilitate hematoma reabsorption. Dexamethasone, a corticosteroid with potent anti-inflammatory effects, was added to reduce cerebral edema and control the inflammatory response. The dosing regimen included Atorvastatin: 10 mg orally once daily.

Dexamethasone: 2.25 mg orally once daily for the first week, 1.5 mg daily for the second week, 0.75 mg daily for the third week, then discontinue. The total dose of the regimen is 31.5 mg. All patients with an initial GCS of 14 or higher and a hematoma volume of less than 30 ml can expect treatment for 24 h under real-time monitoring of vital signs and consciousness assessment. If there is a worsening of consciousness or if a follow-up CT scan after 24 h shows hematoma enlargement, surgery will be performed. If there is no worsening after strict monitoring for 3 days, the monitoring level can be reduced, and the patient can be discharged once a follow-up CT after 7 days shows a reduction in the hematoma (Figure 1B). Patient 2 had an initial GCS score of 13. Due to the guardian's refusal of surgery, the patient entered a conservative treatment process. All patients showed no deterioration in consciousness after admission and underwent CT scan re-examination on the 7th day after injury.

### Clinical outcome

All patients recovered well after conservative treatment and did not require delayed surgical intervention. Three months after the injury, the patients were followed up, and all recovered well without any neurological function loss (Glasgow Outcome Scale = 5).

## Literature review and discussion

We conducted a literature search on PubMed and Google Scholar using the terms “(epidural hematoma) AND ((Conservative treatment) OR (expectant treatment) OR (medication treatment))” (Tables 2, 3). Compared to previous studies on conservative treatment, our therapy may accelerate hematoma absorption and result in full recovery for the patients (Table 3).

**Table 2:** Patient characteristics in the EDH conservative treatment studies.

Author	Research type	Sample size	Hematoma thickness (mm)	Hematoma volume (ml)	Midline shift (mm)	Initial neurological state (GCS)
Present cases	Case report	6	13.5 ± 6.6	15.4 ± 9.3	3.0 ± 3.2	14.3 ± 0.8
Pozzati and Tognetti (23)	Case report	4	NA	“Moderate size”	“Mild shift”	15 ± 0
Edward et al. (24)	Case report	3	NA	NA	NA	15 ± 0
Bullock et al. (25)	Case report	12	NA	26.8	4.7 ± 2.6	13.9 ± 1.1
Pozzati and Tognetti (26)	Case report	22	<15	NA	NA	NA
Servadei et al. (5)	Prospective study	42	<15: 88.1%	NA	<5 92.8%	15: 56.4%
			>15: 11.9%		>5 7.2%	14: 43.6%
Hamilton and Wallace (27)	Prospective study	18	12.6	NA	1.8	13.1
Lahat et al. (28)	Case report	8	NA	NA	NA	14.8 ± 0.4
Wong (29)	Case report	8	17.8 ± 6.5	9.3 ± 5.6	3.9 ± 1.1	13.9 ± 2.6
Mustafa et al. (30)	Retrospective study	14	“Thin (5–12 mm”	NA	“Without mass effect”	15 ± 0
Edson et al. (31)	Case report	3	NA	“Small”	“No mass effect”	15 ± 0
Jamous et al. (32)	Case report	6	16.2 ± 3.0	NA	NA	14.7 ± 0.5
Khan et al. (33)	Retrospective study	17	16 ± 4	NA	5 ± 4	15 ± 0
Maugeri et al. (4)	Case report	1	24	NA	12	14
Basamh et al. (34)	Retrospective study	125	NA	9.4 ± 15.6	0.3 ± 1.2	12.7 ± 3.4
Kun et al. (35)	Case report	3	NA	13 ± 1.6	NA	13.7 ± 0.9
Samantha et al. (36)	Retrospective study	22	NA	9.9 ± 5.4	NA	13–15: 77.3%
						9–12: 18.2%
						3–8: 4.4%
Jamous (37)	Retrospective study	44	7.9 ± 2.4	NA	NA	15: 88.6%
						14: 11.4%
Gok et al. (22)	Retrospective study	143	NA	0–6 years: 10	NA	NA
				6–12 years: 12		
				12–18 years: 15		
				>18 years: 22		

GCS, Glasgow Coma Scale; NA, not available.

**Table 3:** Outcomes of patients in the EDH conservative treatment studies.

Author	Location	No follow-up surgery	Length of stay	Hematoma resolution time	Prognosis (GOS)
Present cases	Supratentorial	100%	NA	2 weeks–2 months	100% GR
Pozzati and Tognetti (23)	Supratentorial	100%	22 ± 7.4 days	1–4 months	100% GR
Edward et al. (24)	Supratentorial	67%	NA	1 month	100% GR
Bullock et al. (25)	Supratentorial	100%	4.5 weeks	7.25 weeks	100% GR
Pozzati and Tognetti (26)	Supratentorial	95.5%	NA	0.5–4 months	100% GR
Servadei et al. (5)	Supratentorial	NA	13.5 days	NA	97.6% GR
Hamilton and Wallace (27)	Supratentorial	94.1%	11.2 days	NA	94.1% GR
Lahat et al. (28)	Supratentorial	100%	7.8 ± 2.5 days	NA	100% GR
Wong (29)	Posterior Fossa	62.5%	NA	NA	62.5% GR
Mustafa et al. (30)	Posterior Fossa	100%	NA	20 days	100% GR
Edson et al. (31)	Posterior Fossa	100%	NA	NA	100% GR
Jamous et al. (32)	Supratentorial	100%	11.7 ± 6.1 days	2–3 months	100% GR
Khan (33)	Supratentorial	88.24%	11.2 ± 7.8 days	NA	100% GR
Maugeri et al. (4)	Supratentorial	100%	14 days	30 days	100% GR
Basamh et al. (34)	Supratentorial 91.2%	88.8%	NA	NA	87.2% GR
	Posterior Fossa 1.6%				
	Occipital 7.2%				
Kun et al. (35)	Posterior Fossa	100%	NA	NA	100% GR
Samantha et al. (36)	Supratentorial 72.7%	100%	3.0 (2.0, 4.0) days^a^	NA	77.3% GR
	Posterior Fossa 27.3%				
Jamous (37)	Supratentorial 93.2%	100%	5.2 ± 3.4 days	50% complete resolution at 1 month	100% GR
	Posterior Fossa 6.8%				
Gok et al. (22)	Supratentorial	77.6%	9 days	NA	81.8% GR

GOS, Glasgow Outcome Scale; GR, good recovery; NA, not available.

^a^ Median (Q1, Q3).

In the present cases, all patients who received a combination of atorvastatin and corticosteroid therapy had faster hematoma absorption and fully recovered their work and social functions within three months. This approach is especially relevant in patients with a Glasgow Coma Scale (GCS) score ≥14 at admission, where spontaneous resolution of the hematoma is possible under careful monitoring (5, 12). Based on the Brain Trauma Foundation guideline (1) and reviewed clinical studies, we conservatively recommend that EDH patients with a GCS ≥ 14 can safely undergo conservative treatment under monitoring (Figure 1B).

In our case series, we observed that patients with EDHs treated conservatively with atorvastatin combined with dexamethasone achieved favorable outcomes without requiring surgical intervention. Notably, all patients demonstrated complete resolution of the hematomas within 2 months, with no neurological deficits at follow-up (GOS = 5). This reinforces previous findings that small EDHs, particularly those without significant neurological deterioration, can be effectively managed conservatively with close observation and serial imaging (4, 5, 12–14).

One of the novel aspects of our study is the use of atorvastatin as an adjunct therapy in the conservative management of EDH. While atorvastatin is widely recognized for its lipid-lowering effects, recent studies have explored its additional benefits, including its anti-inflammatory, anti-apoptotic, and angiogenic properties. In particular, atorvastatin has been shown to promote the resolution of CSDH by enhancing lymphatic drainage and facilitating hematoma absorption. These mechanisms may also extend to EDH, as both conditions share similar pathophysiological features, such as inflammation and impaired lymphatic drainage (8, 10). Dexamethasone is classically used in the treatment of cerebral edema (15), and recent meta-analyses have shown that it reduces mortality from ischemic stroke and cerebral hemorrhage (16). Xuxiang et al. have highlighted the potential benefits of traditional Chinese medicine in enhancing hematoma resolution without surgical intervention (17). In addition to medication, recent studies have found that non-invasive methods such as manual therapy (18), low-intensity ultrasound (19), and phototherapy (20) can promote the brain lymphatic system. These approaches may potentially affect the conservative treatment of EDH (Figure 1C).

However, it is important to note that conservative management remains a highly individualized approach. Patient selection criteria, including age, comorbidities, and the rapidity of hematoma growth, are crucial in determining whether conservative treatment is appropriate. The decision must be made on a case-by-case basis, carefully monitoring neurological status and serial imaging to assess hematoma progression. Gok et al. and Sullivan et al. emphasize the importance of follow-up CT scans in guiding clinical decisions and ensuring that surgical intervention is not delayed in cases of deterioration (21, 22).

While our results are promising, further studies are needed to establish clearer guidelines for using atorvastatin in the conservative management of EDH. Large-scale prospective studies are warranted to validate this treatment regimen's effectiveness and determine the optimal dosage and duration of therapy. Moreover, future research should focus on identifying specific biomarkers or imaging characteristics that could predict which patients are most likely to benefit from conservative treatment, including pharmacological interventions.

## Conclusion

Our findings suggest that atorvastatin, when combined with dexamethasone, may offer a promising alternative to surgery for the conservative management of epidural hematomas. This approach, along with careful monitoring and patient selection, may help expand the non-surgical treatment options available for EDH, particularly in patients who do not exhibit significant neurological deterioration at admission. Further clinical investigations are required to optimize this management strategy and validate its long-term efficacy.

## Data Availability

The datasets presented in this article are not readily available because of ethical and privacy restrictions. Requests to access the datasets should be directed to the corresponding author.
